# Stem cells from human cardiac adipose tissue depots show different gene expression and functional capacities

**DOI:** 10.1186/s13287-019-1460-1

**Published:** 2019-11-29

**Authors:** Carmen Lambert, Gemma Arderiu, Maria Teresa Bejar, Javier Crespo, Maribel Baldellou, Oriol Juan-Babot, Lina Badimon

**Affiliations:** 1 Cardiovascular-Program ICCC, IR-Hospital Santa Creu I Sant Pau, IIB Sant Pau, C/Sant Antoni Ma Claret 167, 08025 Barcelona, Spain; 2 Ciber CV, 28029 Madrid, Spain; 3 Cardiovascular Research Chair UAB, Barcelona, Spain

**Keywords:** Epicardial adipose tissue, Perivascular adipose tissue, Ventricular myocardium adipose tissue, Adipose stem cells, Microvesicles, Angiogenesis

## Abstract

**Background:**

The composition and function of the adipose tissue covering the heart are poorly known. In this study, we have investigated the epicardial adipose tissue (EAT) covering the cardiac ventricular muscle and the EAT covering the left anterior descending artery (LAD) on the human heart, to identify their resident stem cell functional activity.

**Methods:**

EAT covering the cardiac ventricular muscle was isolated from the apex (avoiding areas irrigated by major vessels) of the heart (ventricular myocardium adipose tissue (VMAT)) and from the area covering the epicardial arterial sulcus of the LAD (PVAT) in human hearts excised during heart transplant surgery. Adipose stem cells (ASCs) from both adipose tissue depots were immediately isolated and phenotypically characterized by flow cytometry. The different behavior of these ASCs and their released secretome microvesicles (MVs) were investigated by molecular and cellular analysis.

**Results:**

ASCs from both VMAT (mASCs) and the PVAT (pASCs) were characterized by the expression of CD105, CD44, CD29, CD90, and CD73. The angiogenic-related genes VEGFA, COL18A1, and TF, as well as the miRNA126-3p and miRNA145-5p, were analyzed in both ASC types. Both ASCs were functionally able to form tube-like structures in three-dimensional basement membrane substrates. Interestingly, pASCs showed a higher level of expression of VEGFA and reduced level of COL18A1 than mASCs. Furthermore, MVs released by mASCs significantly induced human microvascular endothelial cell migration.

**Conclusion:**

Our study indicates for the first time that the resident ASCs in human epicardial adipose tissue display a depot-specific angiogenic function. Additionally, we have demonstrated that resident stem cells are able to regulate microvascular endothelial cell function by the release of MVs.

## Background

Cardiovascular diseases (CVDs) are the leading cause of death and disability worldwide, and coronary artery disease makes up the greatest proportion of those deaths. Acute myocardial infarction leads to an irreversible loss of proper cardiac function; implantation of adult stem cells into the ischemic damaged myocardium has been investigated for its potential to repair/regenerate the injured tissue within the infarct zone [1–4].

Adipose tissue (AT) has long been considered an energy storage and an endocrine organ. However, in the last decades, adipose tissue has also been considered a rich source of mesenchymal cells and is currently the focus of interest in the field of inducible spontaneous regeneration and cell therapy. Adipose-derived stem cells (ASCs) are easily obtained, show a strong capacity for ex vivo expansion and differentiation to other cell types (as cardiomyocytes [5] or endothelial cells [6, 7]), release a large variety of angiogenic factors, and have immunomodulatory properties. All these properties have encouraged its use to induce ischemic tissue recovery. However, their clinical use of bone marrow stem cells has been hampered by the recognition of the loss of function effects of aging, cardiovascular risk factors (CVRFs), and metabolic disorders [8]. Indeed, regarding spontaneous endogenous repair, our group has shown that the presence of CVRFs, such as type 2 diabetes mellitus, negatively affects the pluripotency and self-renewal capacities of adipose stem cells (ASCs) [9], and ASCs obtained from obese patients display an impaired angiogenic potential [10, 11]. Indeed, the spontaneous regenerative capacity for ASC self-renewal seems to be regulated by the anatomical WAT depot.

Besides the commonly known CVRFs that are known to elevate the risk of suffering CVDs, excess adiposity is considered a CVRF for CVD [12]. The relationship between obesity and the development of any CVDs lies not only in the amount of fat, but also in its localization. AT is highly heterogeneous tissue due to its different types (white, brown, and “brite”) and its different body localizations (subcutaneous, visceral, epicardial, and perivascular, etc.). Composition, structure, and function of adipose tissue are highly dependent on individual metabolic factors that we are still learning. Ectopic AT covering different organs may have different anatomic and functional characteristics and due to its proximity to various internal organs may exert organ-specific regulatory functions [13].

AT that directly surrounds the heart is known as epicardial adipose tissue (EAT). EAT, a thoracic fat depot that exists on the surface of the myocardium and is contained entirely beneath the pericardium, has acquired increasing interest because of its proximity to the myocardium and coronary arteries and their branches [12]. EAT has been reported to exert an endocrine role by deregulation of adipokine secretion [14, 15] and to have direct effects on local inflammation and coronary atherosclerosis [16–18]. EAT is also a source of ASCs, and it has been reported that these cells may have a higher cardiomyogenic potential as compared to pericardial and omental ASCs subtypes [19].

ASCs not only have interest for their capacity to differentiate into differentiated cells, as cardiomyocytes, but also for their capacity to stimulate angiogenesis promoting progenitor cell differentiation and paracrine proangiogenic and immunomodulatory effects [20]. The proangiogenic effects of conditioned medium derived from ASCs support this paracrine view of stem cell function [4]. Previous studies indicated that microvesicles (MVs) released by ASCs mediate their proangiogenic effects by cell-to-cell communication stimulating formation and stabilization of vessel formation [21]. However, little is known about epicardial ASCs, in particular, those located closely to the vessels [AT around vessels is known as perivascular AT (PVAT)] and those residents in the EAT directly superimposed on the cardiac muscle (VMAT) covering the apex of the heart. The contribution to these two stem cell reservoirs to cardiac cell function and to cardiac endogenous repair processes after an ischemic injury is unknown. As the coronary arteries and their major branches are imbedded in PVAT, this creates a perfect environment for the local interaction between ASCs present in this fat and the coronary vessels it surrounds. Angiogenesis is essential for the repair of wounded or ischemic organs, and insufficient angiogenic switch in an ischemic heart can limit revascularization, healing, and regeneration [22].

Here, we hypothesized that EAT from two different locations in the human heart may contain a repository of stem cells (ASCs) with different pro-angiogenic functions.

## Materials and methods

### Sample collection/patients

VMAT and PVAT were obtained from the excised hearts of 30 patients undergoing cardiac transplant surgery at the Hospital de Sant Pau i la Santa Creu (Table 1). Only samples from 3 patients were excluded, 2 of them due to internal cell contamination and the other 1 due to endocarditis. Tissue was obtained with informed consent of patients. The protocol was approved by the Research Ethics Committee of our hospital and was conducted in accordance with the Declaration of Helsinki. Patients until the moment of transplant surgery were treated as recommended by clinical practice guidelines. Left anterior descending (LAD) coronary arteries were collected and PVAT isolation. Arteries were fixed in 4% paraformaldehyde and stored at − 80 °C embedded in OCT until histological analysis.

**Table 1:** Clinical characteristics of the study patients

Variables	Patients
*N*	30
Age (years)	54 ± 3
Sex (M/W)	19/11
BMI (kg/m^2^)	27.4 ± 1.0
Obesity (%)	33.3
Overweigh (%)	20
Preserved FE (%)	20
CVRFs	2 ± 1
Smoking (%)*	23
HTA (%)**	47
DM (%)**	17
DLP (%)**	57

Values are expressed as mean ± SD or as percentages, when indicated

*CVRFs* cardiovascular risk factors, *HTA* hypertension, *DM* diabetes mellitus, *DLP* dyslipidemia

*Five years ex-smokers were considered non-smokers

**Treated as per guidelines

Simultaneously, epicardial adipose tissue directly overlying the ventricular myocardium at the apex of the heart (VMAT) was collected. All studies were run in parallel with samples from both locations from the same patients. Both VMAT and PVAT were carefully dissected and frozen in liquid N_2_ and kept at − 80 °C until used or placed in tubes with 10 mL of Dulbecco’s modified Eagle’s medium (DMEM; Gibco, Life Technologies, Grand Island, NY, USA) supplemented with 1% penicillin/streptomycin (P/S, Gibco, Life Technologies, Grand Island, NY, USA) to be immediately processed to isolate ASCs.

### ASC isolation and characterization

**Fig. 1:**
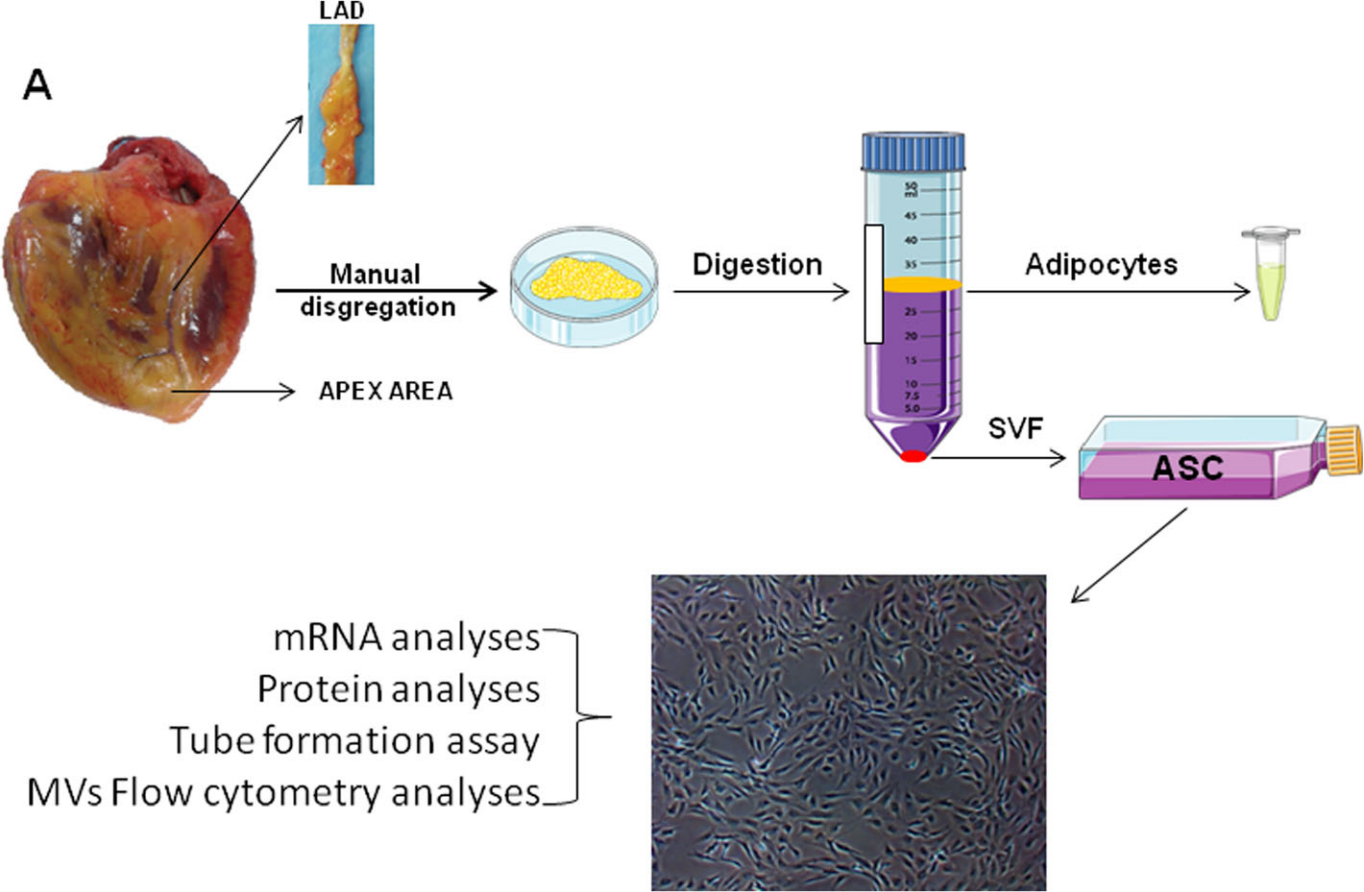
Study design. Ventricular myocardium adipose tissue (VMAT) and perivascular adipose tissue (PVAT) were obtained during heart transplant surgery. ASCs were immediately isolated and phenotypically characterized by flow cytometry

PVAT and VMAT tissues were washed with sterile phosphate-buffered saline (PBS) supplemented with 1% of P/S. Tissue was digested into a type I collagenase solution (1 mg/mL; Sigma-Aldrich, St. Louis, MO, USA) and incubated for 1 h in a 37 °C pre-warmed orbital shaker. Collagenase activity was neutralized with the same amount of fetal bovine serum (FBS; Biological Industries, Kibbutz Beit-Haemek, Israel) and the suspension filtered through a 100-μm strainer to eliminate remaining tissue fragments. Then, the suspension was centrifuged at 1200 rpm for 10 min to separate the adipocytes and to obtain the stromal vascular fraction (SVF). Isolated SVF cells were counted and separated into two aliquots to be analyzed by flow cytometry and to be plated onto 25-cm^2^ culture flasks. After 24 h, non-adherent cells were removed and the medium replaced. Cells were expanded in a humidified environment at 37 °C with 1% O_2_ and 5% CO_2_ and maintained at sub-confluent levels prior to phenotypic profile analysis. The cells were characterized as stem cells by using the following criteria: adherence to plastic, cell surface antigen phenotyping, and differentiation into multiple cell lineages. All analyses were performed between passages 3 and 4. A simplified scheme of the procedure is shown in Fig. 1.

For cell cytometry characterization, cell surface antigen phenotyping was performed on SVF and ASCs obtained from SVF at passage 3 (P3). The following cell surface epitopes were marked with anti-human antibodies: CD105, CD44, CD29, CD90, CD73, CD45, and CD14. Cells (1 × 10^5^) at passage 3 or 1 mL of the SVF was suspended in flow cytometry buffer (PBS, 0.1% BSA, 0.1% sodium azide) and incubated for 30 min at 4 °C with the corresponding antibodies. After that, the reaction was stopped by adding 500 μL of flow cytometry buffer or 250 μL of Quicklysis reagent (Cytognos) in the case of the SVF. Quicklysis was incubated for 15 min at room temperature to eliminate erythrocytes, and the reaction was stopped by adding 250 μL of flow cytometry buffer.

Cellular events (at least 30,000 in the case of ASCs and between 10,000 and 60,000 in the case of the SVF) were acquired and analyzed by fluorescence-activated cell sorting using Coulter EPICS XL flow cytometer (Beckman Coulter) running Expo32 ADC XL 4 color software (Beckman Coulter).

Adipogenic and osteogenic differentiation was also evaluated for further characterization as previously described [11]. Briefly, cells were seeded in 6-well and 24-well plates for the differentiation assay and for RNA isolation or cell staining, respectively. Cells were allowed to grow to sub-confluence (approximately 80% of confluence) and then allowed to differentiate for 21 days with the corresponding conditional medium or normal medium without supplements as a negative control (Additional file 1: Table S1). After 21 days, RNA from the differentiated cells was isolated, and also, cells were stained with Oil Red to detect adipogenic differentiation and Alizarin red to detect osteogenic differentiation.

### Growth kinetics of ASCs

To determine the growth kinetics of ASCs, 2 × 10^4^ cells per well were seeded in ten wells of 6-well plates. Cells from two duplicate wells were harvested and counted every other day for 10 days (cells were counted on days 2, 4, 6, and 10). ASC number was plotted against the number of days cultured. Population doubling time (PDT) was calculated using the formula PDT = Te/[(log N2 − log N1)/log2], where Te is time (days) in the exponential growth phase, *N*1 is the number of cells at the beginning, and *N*2 is the number of cells at the end of the experiment.

### MTS viability/proliferation analysis

Cell proliferation was determined by 3-(4,5-dimethylthiazol-2-yl)-5-(3-carboxymethoxyphenyl)-2-(4-sulfopheny)-2H-tetrazolium (MTS) assay (CellTiter 96 Aqueous One Solution cell proliferation assay kit; Promega). For this assay, 15 × 10^3^ of ASCs or 1 × 10^4^ human microvascular endothelial cells (HMEC-1) were seeded in triplicates into 96-well plates. ASCs were cultured for 24 h and then treated with 10 μL of MTS. HMEC-1 were cultured for 24 h then treated with ASC-derived MVs for other 24 h, and finally, 10 μL of MTS per well was added and incubated for an additional 2 h, while MTS tetrazolium is reduced to formazan (490 nm absorbance) by the metabolically active cells. The absorbance was then quantified with the spectrophotometer Spectramax 250 and analyzed with the SoftMax software (Molecular Devices). Formazan production was directly related with the number of cells alive in the culture.

### Tube-like formation assay

Tube-like formation assay in three-dimensional basement membrane culture was employed to assess the angiogenic potential of ASCs. Briefly, 1 × 10^5^ cells were seeded in a Matrigel-coated plate and incubated with endothelial cell growth medium (EGM-2 BulletKit, Lonza, USA) for 24 h to allow the formation of tube-like structures. The total tube-covered area was quantified using ImageJ software (National Institutes of Health, USA).

### ASC-conditioned medium collection

Cells at passage 3 or 4 were allowed to grow until reaching sub-confluence, then cells were washed exhaustively with PBS to remove FBS and serum-free medium was added. After 48 h, the ASC-conditioned medium was collected and centrifuged at 1200 rpm for 10 min to remove cellular debris and kept at − 80 °C until used.

### Microvesicle isolation

ASC-derived microvesicles (MVs) were isolated by ultracentrifugation of P3 cell supernatants as previously described [23]. Briefly, fresh supernatants were firstly centrifuged at 900*g* for 15 min to eliminate cell debris and then at 20,000*g* for 45 min to isolate the MVs as a pellet. MV concentration was determined by flow cytometry. For that, MVs were washed with a PBS-citrate buffer and centrifuged again at 20,000*g* for 30 min. MVs were extracted with PBS-citrate buffer and incubated with Annexin V (CF Blue ANXV, Immunostep) and anti-TF antibody (FITC-conjugated 4508CJ, Sekisui). Samples were then diluted with Annexin V binding buffer (BD Bioscience) to stop the reaction and then analyzed on a FACSCantoll™ flow cytometer (BD Bioscience). The formation of MVs after cell activation is initiated by the increase of intracellular calcium resulting in an elementary rearrangement of the phospholipid asymmetry with translocation of phosphatidylserine from the inner to the outer surface leaflet of the plasma membrane as a consequence of activation of scramblase and floppase/ABC1 and inhibition of translocase/flippase activities. The presence of phosphatidylserine at the surface of the MP membrane enables the use of the Annexin V for MVs detection by flow cytometry. MVs’ gate limits were established following two criteria: (1) calibration using a Flow-Check Size Range Calibration Kit (Polysciences) and (2) using an in vitro platelet-derived microparticle population as a positive control. The lower detection limit was placed as a threshold above the electronic noise of our flow cytometer.

### Cell migration

HMEC-1 were used for the cell migration assays. Briefly, 2.3 × 10^4^ eASCs were seeded into 100-mm dish and cultured with MCDB 131 medium supplemented with 10% of FBS for 48 h to allow cells to secrete MVs. The day after, 2.5 × 10^5^ HMEC-1 cells were seeded into a Culture-Insert 2 well dish (Idibi) and kept with MCDB 131 supplemented with 10% of FBS overnight. MVs from the eASC supernatant (eMVs) were isolated, and before performing the experiment, the insert was removed by using sterile tweezers and the dish washed with PBS to remove cell debris. Cells were treated with 600 μL of (A) conditional medium from ASCs after 48 h of culture, (B) conditional medium from ASCs without eMVs after 48 h of culture, and (C) eMV-enriched medium after 48 of culture. In all conditions, medium was supplemented with 2% of FBS. Cell migration and wound repair were controlled every 2 h for 10 h. Wound areas were analyzed using ImageJ software. Protein, RNA, and microRNA were isolated from the ASCs; microRNA from the eMVs and RNA; and microRNA from the HMEC-1 cells after 24 h of cell migration.

### Gene expression analysis

Total RNA was isolated from ASCs in silica membrane columns with the Qiagen RNesasy Mini Kit (Qiagen) according to the manufacturer’s instructions.

MirVana miRNA isolation kit (Life Technologies) was used to extract microRNA from the cells, and miRNeasy Serum/Plasma Kit for the microRNA isolation from MVs, according to the manufacturer’s instruction.

RNA and microRNA quantity was determined with Nanodrop ND-1000 spectrophotometer (Nanodrop Technologies). Isolated total RNA was reverse-transcribed into cDNA using the High Capacity cDNA Archive kit (Applied Biosystems, Foster City, CA, USA) and microRNA with the TaqMan advanced miRNA assay (Life Technologies). Gene expression analysis was carried out by quantitative PCR using TaqMan® Gene Expression assays (Applied Biosystems; Additional file 1: Table S2) and the Applied Biosystems Prism 7900HT Sequence Detection System (Applied Biosystems) according to manufacturer’s instructions. Gene expression data are expressed as target gene mRNA expression relative to the correspondent housekeeping gene expression.

### Western blot analysis

Protein was extracted from total cell lysates by using RIPA buffer (50 mM Tris-HCl pH 8, 150 mM NaCl, 1% NP-40, 0.5% sodium deoxycholate, 0.1% SDS) or from 48 h cell supernatant. Protein concentrations were measured with the Pierce BCA Protein Assay Kit (ThermoScientific). Twenty-five micrograms of protein was resolved by 1-DE under reducing conditions onto 10% SDS-PAGE gels and electrotransferred to nitrocellulose membranes. After blocking for non-specific binding with 5% of bovine serum albumin (BSA; MP Biomedical) or Blotto, the membranes were incubated with primary antibodies, including TF (4501-Sekisui Diagnostics), endostatin (ab109660-abcam), and VEGF (ab51745-abcam). Band detection was performed using a chemiluminiscent substrate dye (SuperSignal® West Dura Extended Duration Substrate, Thermo Scientific, Waltham, MA, USA) and a molecular imager ChemiDoc XRS System, Universal Hood II (BioRad, Hercules, CA, USA). Band quantification was performed with Image Lab 4.0 software (BioRad Laboratories, Hercules, CA, USA). Protein load was normalized with total protein staining, and normalization between different membranes was performed with a common pool in every gel, as previously described [24].

### Statistical analysis

Non-parametric Wilcoxon or Mann-Whitney analyses were performed to analyze the differences between tissues. Data are expressed as mean ± SEM unless stated. The level of significance was set at *P* < 0.05. All analyses were conducted with StatView software.

## Results

### Sample characterization

**Fig. 2:**
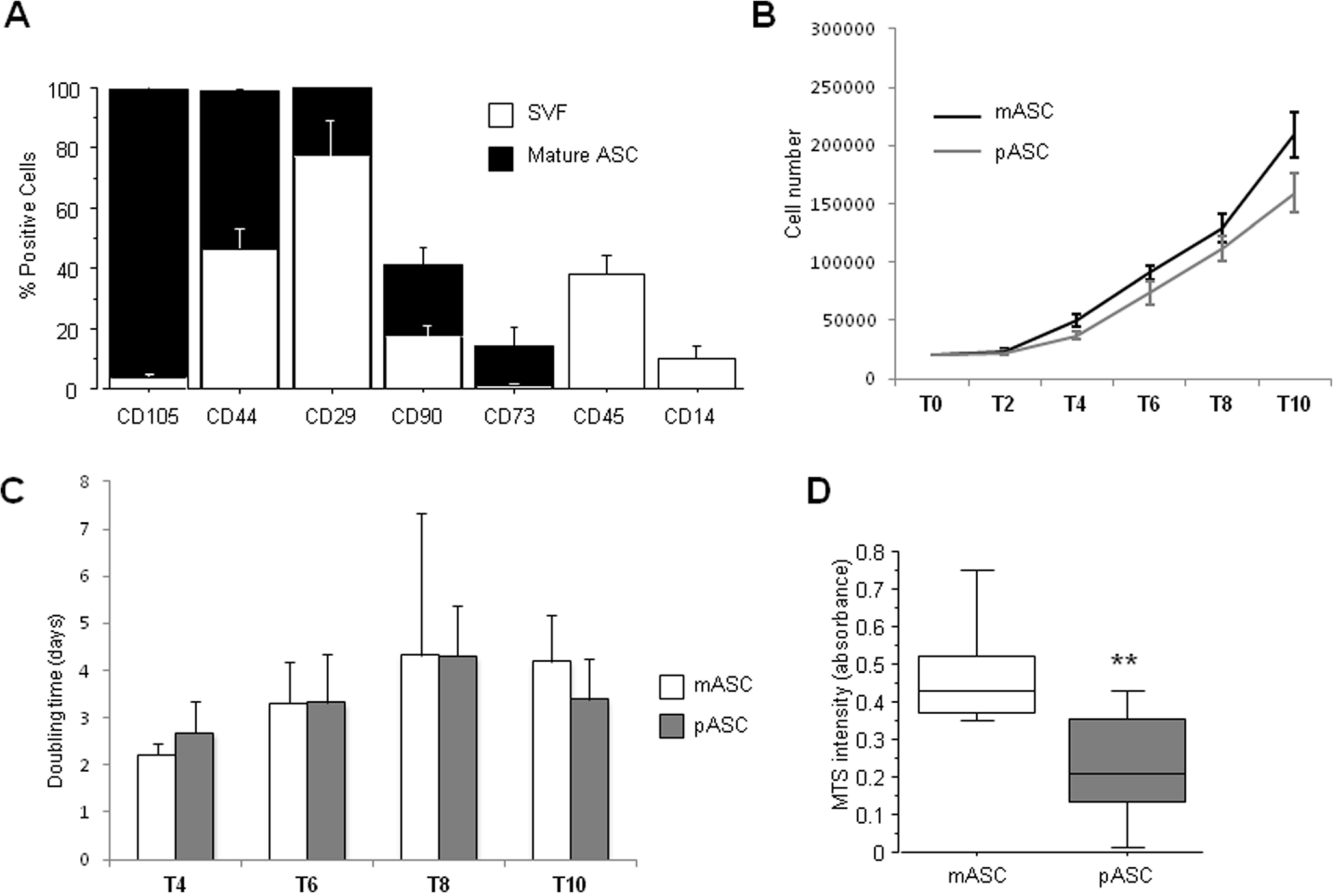
ASC characterization. **a** Flow cytometry analysis. Surface mesenchymal stem cell markers were analyzed from the stromal vascular fraction (SVF) and from cells at passage 3. Data is presented as percentage of positive cells. **b** Growth curves of mASC and pASV. Cells were counted every day after and plotted in a line curve graph. **c** Population doubling time (PDT) of mASC and pASC measured every 2 6 for 10 days. **d** MTS viability assay (**P < 0.01)

Heart adipose tissue and LAD coronary arteries were collected at the operating room during heart transplantation surgery. LAD coronary arteries were classified according to the AHA classification from intimal thickening (IT) to type VI and total occlusion (TO) (Additional file 1: Figure S2A-H). ASCs were isolated immediately after tissue dissection, harvested, and characterized by their adherence to plastic, their ability to differentiate into multiple cell lineages, and the expression of different cell surface markers. ASCs were positive to CD29, CD44, CD73, CD90, and CD105, and characteristic mesenchymal stem cell surface markers, and negative for the hematopoietic marker CD45 and the monocyte marker CD14 (Fig. 2a). These results show that the adipose tissue covering the human heart is a repository for stem cells.

### Proliferation and viability of ASCs from the different adipose tissue depots

The proliferative rate of ASCs obtained from VMAT (mASCs) was always higher than that of ASCs obtained from PVAT (pASCs) (Fig. 2b); however, the main change was observed during the first 4 days; thus, the calculated doubling time of mASCs and pASCs was almost the same during days 6 and 8 (Fig. 2c). Interestingly, the cellular viability MTS assay demonstrated a significantly higher viability in mASCs than in pASCs (*P* = 0.001; Fig. 2d) after 24 h in culture.

### In vitro angiogenic-related gene expression of apex EAT- and PVAT-derived ASCs

**Fig. 3:**
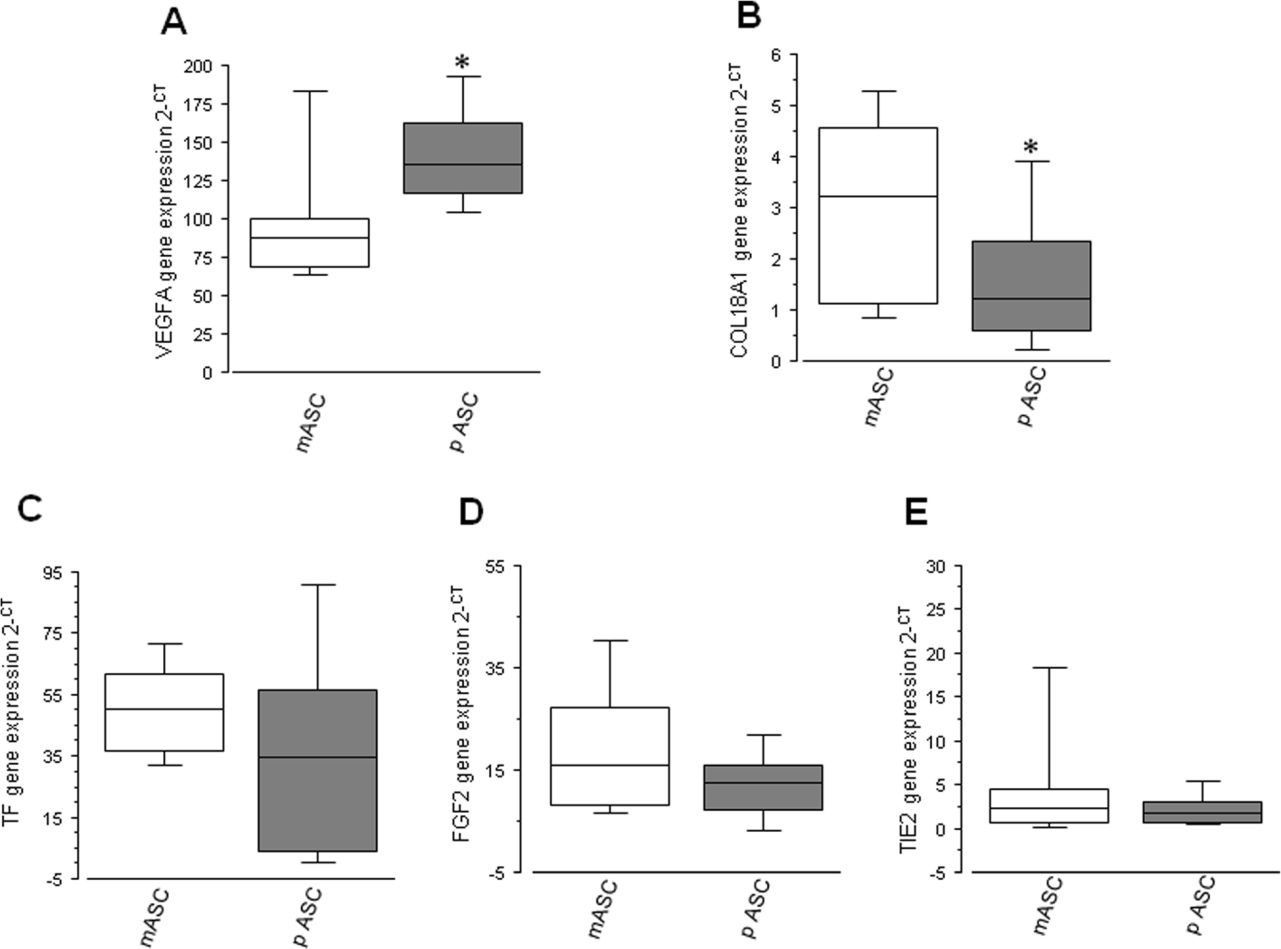
Boxplot diagram showing ASC genomic profile. **a** Differential protein expression of VEGFA in mASC and pASC. **b** Differential protein expression of COL18a1 in mASC and pASC. **c** Differential protein expression of TF in mASC and pASC. **d** Differential protein expression of FGF2 in mASC and pASC. **e** Differential protein expression of TIE2 in mASC and pASC (*P < 0.05; **P < 0.01)

In order to evaluate the angiogenic potential of mASCs and pASCs, different angiogenic-related genes were measured. pASCs showed a significantly higher mRNA expression of VEGFA compared with apex-derived ASCs (*N* = 15; *P* = 0.04; Fig. 3a) and a significantly reduced mRNA level of COL18a1 (*N* = 15; *P* = 0.02; Fig. 3b). Instead, pASCs and mASCs showed similar mRNA expression levels of TF (*N* = 12; *P* = 0.24; Fig. 3c), FGF2 (*N* = 6; *P* = 0.46; Fig. 3d), and TIE2 (*N* = 7; *P* = 0.31; Fig. 3e).

Protein levels of TF and endostatin were also measured by western blot, and a non-significant higher level of TF on pASC compared to mASC (*P* = 0.093; Additional file 1: Figure S2A) was observed. Endostatin levels were measured both in cells and secretome, but no differences were observed between tissues (Additional file 1: Figure S2B-C).

The ability of both ASCs to form tube-like structures was investigated. Both mASCs and pASCs had a similar capacity to induce tube-like formation in Matrigel cultures (Additional file 1: Figure S3A and B, *N* = 9; *P* = 0.21). The ability to form tubes by pASCs derived from LADs with mild atherosclerosis (*N* = 4; IT to grade III) and pASCs derived from LAD with severe atherosclerosis (*N* = 5; grade IV occlusion to TO) was not significantly different (Additional file 1: Figure S3C).

### Effect of obesity and diabetes on eASCs released microvesicle function

**Fig. 4:**
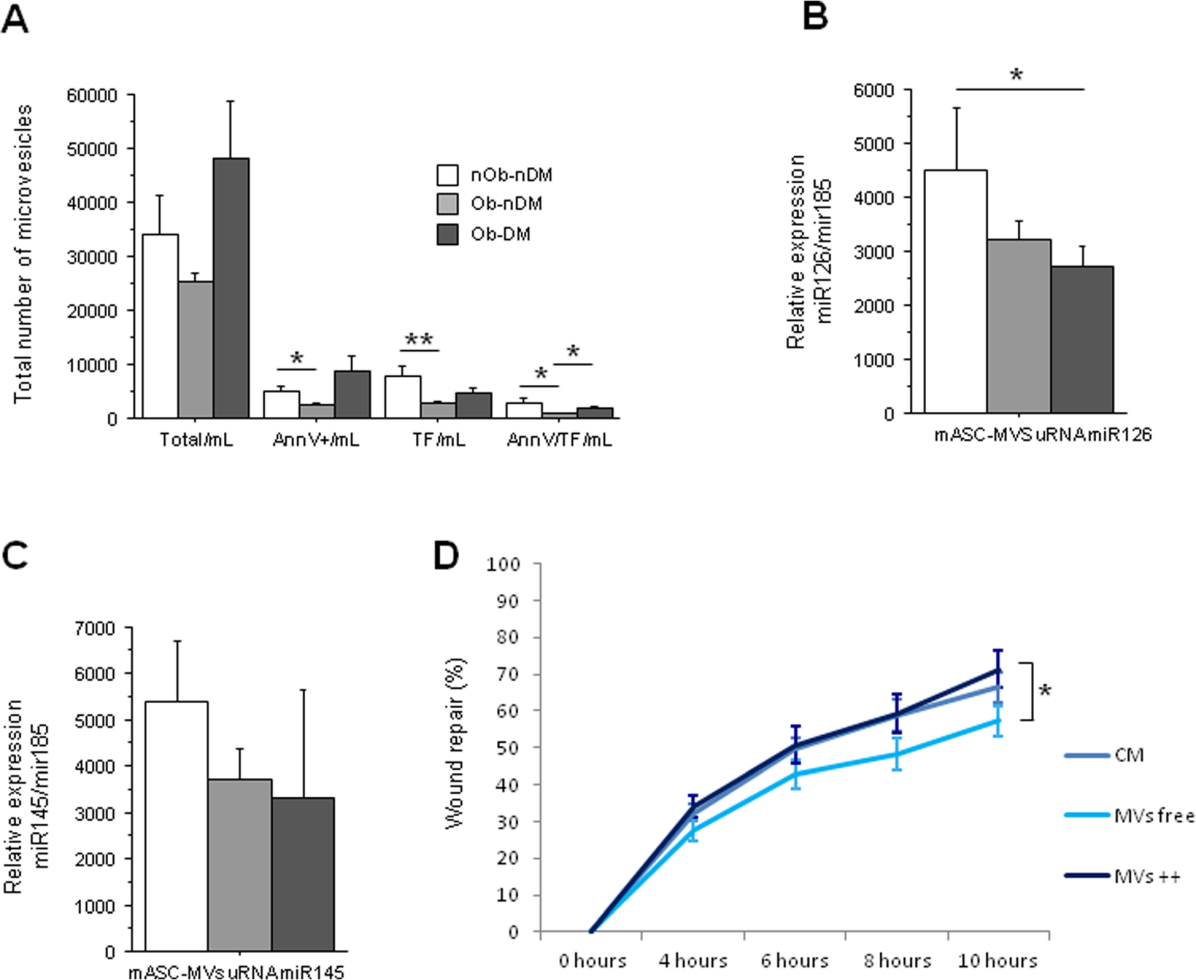
mASC microvesicle (mMV) secretion. **a**–**c** Influence of obesity and diabetes on eASC-derived MVs. **a** Number of mMVs measured by flow cytometry (total, Annexin V-positive, TF-positive, and Annexin V/TF-positive mMVs). **b** miR126 relative gene expression. **c** miR145 relative gene expression. **d** Healing rate line diagram of HMEC-1 cells treated with mASC total medium, mMV-depleted medium, or eMV-rich medium (*P < 0.05; **P < 0.01)

Because of the impact of obesity in adipose tissue accumulation and fat characteristics, we investigated whether mASC-derived microvesicles (mMVs) could exert a regulatory effect on human microvascular endothelial cell proliferation. First, we observed that mASCs from obese non-diabetic patients release less mMVs than those ASCs from obese diabetic patients (*P* = 0.06; Fig. 4a). Interestingly, obese and diabetic mASC-released mMVs have significantly higher levels of Annexin V than those released by non-obese non-diabetic patients (*P* = 0.03; Fig. 4a). Annexin V binds to phosphatidylserine that is usually exposed in the outer leaflet of the plasma membrane of cells that are activated or apoptotic and released in the microvesicles. Next, we analyzed whether these MVs were also positive for TF, because previous studies in our group have described that TF-rich microvesicles increase angiogenesis in ischemic tissue [25]. The mMVs from mASCs of non-obese and non-diabetic patients (total or Annexin V positive) contain significantly higher TF amount than those mASCs from obese non-diabetic patients (*P* = 0.004 and *P* = 0.02, respectively; Fig. 4a). mMVs from mASCs of obese non-diabetic patients also contain less TF than mMVs from mASCs of obese diabetic patients (*P* = 0.02; Fig. 4a).

We then analyzed the microRNA (miRNA) composition of those eMVs. We analyzed two different miRNAs that have been implicated in ASCs and the angiogenic process as miRNA 126-3p and miRNA 145-5p [26, 27]. We found that mMVs from mASCs of non-obese non-diabetic patients had a significantly higher content of miRNA 126-3p than those mMVs from mASCs of obese diabetic patients (*P* = 0.05; Fig. 4b). Non-significant change was observed on miRNA 145-5p (Fig. 4c).

To evidence the effects of mASC-secreted mMVs on human microvascular endothelial cell function, we first performed a MTS viability assay and no significant effects on cell viability after treatment with mMVs was observed; however, HMEC cells treated with mMVs from non-obese non-diabetic patients showed a non-significant improvement on HMEC viability (Additional file 1: Figure S4A).

The effect of mASC-secreted mMVs on HMEC-1 migration showed that, after 10 h of incubation, those wounds treated with mMV-free secretome were significantly less able to repair the wounded area compared with those treated with mMV-rich medium (*P* = 0.05; Fig. 4d), with the highest effect seen when HMEC-1 cells were treated with mMVs released by mASC from obese diabetic patients (Additional file 1: Figure S4B-D).

**Fig. 5:**
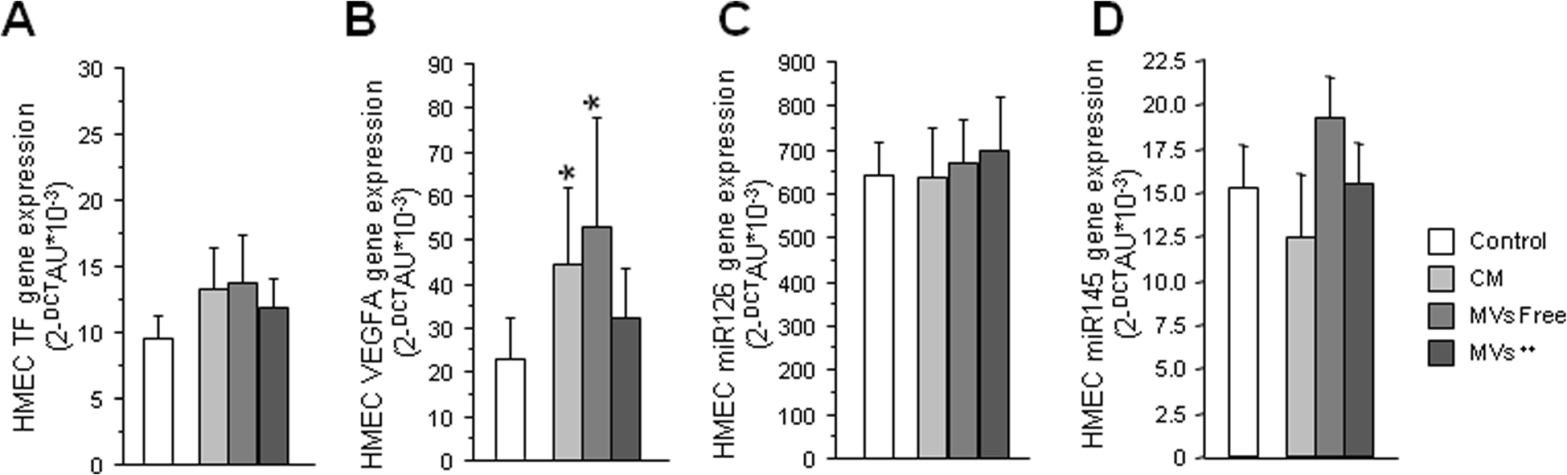
Effect of mASC-derived MVs on HMEC-1. Genomic changes of HMEC-1 cells produced by the treatment with mASC total medium, mMV-depleted medium, or mMV-rich medium. **a** HMEC-1 VEGFA gene expression. **b** HMEC-1 TF gene expression. **c** HMEC-1 miR126 gene expression. **d** HMEC-1 miR145 gene expression (*P < 0.05)

To evidence the effect of mMVs on receptor HMEC-1 cells, we analyzed the mRNAs and the miRNAs expressions in the receptor cells that had been incubated with the supernatants of EAT-ASCs. We observed no change on TF expression (Fig. 5a) nor in miRNA 126-3p (Fig. 5c) and miRNA 145-5p (Fig. 5d), but a significant increase on VEGFA (Fig. 5b) expression induced by both total CM (*P* = 0.01) and mMV-free medium (*P* = 0.01), implying effects independent of MVs but associated with soluble factors.

These results indicate that obesity and diabetes impact on the pro-angiogenic capacity of resident stem cells and explain the low spontaneous regenerative efficacy of stem cells in patients with metabolic syndromes.

## Discussion

The EAT has emerged as a depot with a distinctive biological significance in cardiac function. Moreover, it is a reservoir of ASCs, with yet undefined effects on the potential regeneration of ischemic tissue. Here, we show that ASCs contained in different EAT locations obtained from patients with advanced heart failure requiring transplant have distinct functional properties.

The relationship of the adipose tissue with CVD has been widely studied [28, 29]. Around 80% of the heart is covered by visceral ectopic adipose tissue depots, which in lean subjects is considered cardioprotective because of the secretion of anti-inflammatory and anti-atherogenic adipokines. However, excess and thickness of fat around the heart, usually measured by imaging techniques, have been associated with CVD [16, 30]. Although imaging has brought a growing interest in the presence of heart EAT, it has not been possible to functionally study this adipose tissue as widely as it has been done with other more accessible depots [31].

The ectopic visceral adipose tissue covering the heart can be sub-classified by location and as perivascular fat and fat directly overlying the myocardium, with blood supplies directly derived from the coronary arteries and their branches [32, 33]. The function of the stem cells found in these adipose tissue depots has not been studied in-depth. This study has focused on the identification, isolation, and comparison, for the first time, of the ASCs covering the LAD (PVAT) and the ASCs covering the ventricular myocardium at the apex (VMAT) of hearts explanted during heart transplant operations. These ASCs were characterized by their adherence to plastic as well as by the expression of different stem cell markers (CD90, CD105, CD44, CD29, and CD73) and the absence of the leukocyte marker CD45 and the innate immune system marker CD14. Thus, both fat locations have a significant amount of resident stem cells.

We have observed that mASCs have a higher proliferative rate and growth kinetics than pASC, which might suggest that mASCs are cells with an active participation in local regenerative activity and homeostasis. Due to the angiogenic activity of adipose tissue [34], we investigated the genomic angiogenic profile of these cells. Even though there are many proteins involved in the angiogenic process, VEGF is undoubtedly the most important angiogenic factor. It is a potent mitogen for endothelial cells (ECs), involved both in the development of the vascular system and the induction and maintenance of the neovasculature in tumors. VEGF binds to any of the tyrosine kinase receptors (VEGFR1 or VEGFR2), which induce the phosphorylation of the tyrosine residues, thus activating intracellular signaling pathways involved in EC proliferation, migration, and survival [35, 36]. On the other hand, COL18A1 is a gene that encodes the anti-angiogenic protein endostatin in its C-terminal domain [37]. Endostatin inhibits EC proliferation and migration and induces EC apoptosis by blocking the binding of VEGF with the tyrosine receptor KDR (VEGFR-2) [38]. We found that pASCs express a higher amount of VEGFA, as well as a reduced expression of COL18A1, compared with the mASCs, suggesting a coordinated regulation of angiogenesis through these two proteins by EAT-derived ASCs, which is increased on pASC, in direct contact with the LAD compared with mASC. By contrast, no differences by location were achieved in other genes as TF, TIE2, and FGF-2. The role of these genes in angiogenesis is well established [39–41]; in fact, FGF-2 induces the formation of new capillary-like tubules on HMEC cells [42]. However, in line with our results, in previous studies, no differences were observed when comparing the expression of FGF-2 in ASC from different sources, but changes on these proteins were obtained as a result of the age of the donor [43]. TIE-2 is related with the ANGPT system [44]; thus, a deep study on this pathway is needed for the better understanding of this new source of epicardial ASC.

Few studies have analyzed the presence and function of heart adipose tissue-derived stem cells, neither in animal [45, 46] nor in human [19] models, and in none of them, their angiogenic potential has been analyzed and compared. Previous studies in our group have shown how ASCs derived from the subcutaneous adipose tissue, independently of the BMI, have no ability to form tubes in a Matrigel-based experiment [11], but here, we demonstrate for the first time that both mASCs and pASCs, when seeded in a 3D Matrigel surface and cultured with EGM-2 medium, are able to form capillary-like ring structures, independently of cardiac fat location and of atherosclerosis severity in the LAD indicating a proangiogenic function. More studies are needed to analyze the influence of different co-morbidities on heart adipose tissue and its derived ASC.

Angiogenesis is mediated not only by VEGFA [47] and COL18A1 [48], but also by many other genes, such as fibroblast growth factor (FGF), angiopoietin I and II (ANGPT I-II) [41], and NOTCH [49] among others, and target cells include endothelial cells (EC) and vascular smooth muscle cells (VSMCs) [50, 51] were involved.

In fact, a coordinated reaction between different cellular types and metabolic factors is needed for the correct development of the angiogenic process [52]. It would also be of great interest to analyze the adipokines secreted by these EATs and resident ASCs to further characterize both epicardial heart depots and their potential implication on myocardial self-regeneration.

Spontaneous regeneration of damaged tissue may be accomplished not only by ASC differentiation and also by ASC-released microvesicles (MVs) and exosomes [53, 54]. MVs are small particles with a diameter ranging from 0.10 to 1.00 μm released by many cell types, including ASCs [55]. The potential of MVs released by ASCs to improve HUVEC migration, proliferation, and angiogenesis has been reported [56, 57]. Many studies support the concept of a proangiogenic effect of MVs depends on their composition, conferring to them a dynamic storage pool of bioactive molecules that play essential roles in cell-cell crosstalk. In this way, MVs can provide intercellular communication by the delivery of miRNAs to influence transcription and altering genetic processes. miRNA126-3p is one of the more important miRNAs in angiogenesis [58], and it has been described to be present in MVs. In line with these results, we have also demonstrated that heart mASCs from non-obese and non-diabetic heart failure patients release mMVs, rich in miRNA126-3p, that improve microvascular endothelial cells (HMEC-1) viability and are more effective than mASC-conditioned medium (CM), or mMV-depleted medium in inducing angiogenesis. mMVs also increase HMEC-1 migration rate, independently on the presence of CVRFs, and HMEC-1 wounds in vitro are closed faster when treated with mMVs from obese diabetic patients. In this regard, it has been reported that MVs released from subcutaneous ASCs of obese subjects have impaired pro-angiogenic potential and harbor reduced VEGF, MMP-2, and miR-126 content than those released by ASCs of non-obese subjects, reducing their capability to promote EC migration and tube-like structure formation [26]. In addition, previously, we have found that TF-rich MVs increase neovascularization in ischemic tissue [23], and here, we observed that mASCs have high levels of TF that may induce the angiogenic properties of these MVs and are decreased in obese and diabetic patients. HMEC-1 treated with eASC supernatant (CM, MV-free medium or MV-rich medium) increases the expression of VEGFA, suggesting a potential way of action of ASC extracellular particles (not only MVs, but also exosomes and other secreted factors). TF induces angiogenesis by ETS1 transcription factor [59], and VEGF is a target gene of ETS1 [60]. However, more experiments on this field should be carried out to elucidate the precise mechanism of action of each different extracellular particle depending on ASC origin.

### Limitations

As a limitation, we have to report the low number of cases in this study, but the number of heart transplants in one single hospital is low. Organ donations are distributed among patient waiting lists in many different hospitals. The methods and technologies reported here have to be performed immediately with hearts directly obtained by research investigators waiting at the surgical room to receive the excised heart. At the annex of the operating room, the hearts are sampled and taken from the operating room within minutes in the appropriated buffers to the cell biology laboratory for further processing. Therefore, only in-hospital transplants could be used. Many other genes and pathways would be analyzed if more hearts were available.

## Conclusions

In the present study, we demonstrate for the first time that hearts from patients with advanced heart failure requiring transplant have ASCs in their different heart adipose tissue depots and that these ASCs have distinct functional properties. Additionally, we have demonstrated the potential role of mASC-derived microvesicles on endothelial cell migration that could represent a promising strategy to stimulate spontaneous regeneration and repair in rarefaction areas of the damaged myocardium. Tissue activation and mobilization of resident ASCs could be a potential means to reduce the progression of disease in failing hearts.

## Supplementary information

**Additional file 1: Figure S1.** A-H) LAD coronary arteries classification. According to the American heart association, LAD coronaries were classified from intimal thickening (IT) to type VI and total occlusion (TO). **Figure S2.** Change in protein levels measured by western blot. A) Tissue factor protein expression in mASC compared with pASC. B) Endostatin protein expression in mASC compared with pASC. C) Endostatin protein expression in mASC supernatant compared with pASC supernatant. **Figure S3.** Tube-like formation assay. A) Representative image of 3D-matrigel-tube formation in mASC and pASC. B) Box plot comparing the tube length from the mASC and the pASC. C) Bar chart comparing the tube formation potential of pASC from non-occluded LAD coronaries and from occulted LAD coronaries. **Figure S4.** Influence of different CRF on mMVs proliferation potential. A) MTS viability assay. Change on HMEC-1 viability when treated with mMVs from patients with different number of CVRFs. B) Healing rate line diagram of HMEC-1 cells treated with mASC total medium, mMVs depleted medium or mMVs rich medium from non-obese and non-diabetic patients. C) Healing rate line diagram of HMEC-1 cells treated with mASC total medium, mMVs depleted medium or mMVs rich medium from obese and non-diabetic patients. D) Healing rate line diagram of HMEC-1 cells treated with mASC total medium, mMVs depleted medium or mMVs rich medium from obese and diabetic patients (*P=0.05). **Table S1.** Adipogenic and osteogenic conditional medium composition. **Table S2.** Taqman gene expression assays.

## Data Availability

All data generated and materials are included in the manuscript.

## Abbreviations

ASCs: Adipose stem cells
CM: Conditional medium
CVD: Cardiovascular disease
CVRF: Cardiovascular risk factor
eASCs: Epicardial adipose stem cells
EAT: Epicardial adipose tissue
HMEC-1: Human microvascular endothelial cells-1
LAD: Left anterior descendent
MVs: Microvesicles
mASCs: Ventricular myocardium adipose stem cells
mMVS: mASC-derived microvesicles
pASCs: Perivascular adipose stem cells
PVAT: Perivascular adipose tissue
SVF: Stromal vascular fraction
VMAT: Ventricular myocardium adipose tissue
VSMC: Vascular smooth muscle cell
