# Supplementary material for: Stem cells from human cardiac adipose tissue depots show different gene expression and functional capacities
Source: Stem Cell Res Ther. 2019 Nov 29;10:361. doi: 10.1186/s13287-019-1460-1 (PMC6884762; doi:10.1186/s13287-019-1460-1)
Supplement: Supplementary file 1 — Additional file 1: Figure S1. A-H) LAD coronary arteries classification. According to the American heart association, LAD coronaries were classified from intimal thickening (IT) to type VI and total occlusion (TO). Figure S2. Change in protein levels measured by western blot. A) Tissue factor protein expression in mASC compared with pASC. B) Endostatin protein expression in mASC compared with pASC. C) Endostatin protein expression in mASC supernatant compared with pASC supernatant. Figure S3. Tube-like formation assay. A) Representative image of 3D-matrigel-tube formation in mASC and pASC. B) Box plot comparing the tube length from the mASC and the pASC. C) Bar chart comparing the tube formation potential of pASC from non-occluded LAD coronaries and from occulted LAD coronaries. Figure S4. Influence of different CRF on mMVs proliferation potential. A) MTS viability assay. Change on HMEC-1 viability when treated with mMVs from patients with different number of CVRFs. B) Healing rate line diagram of HMEC-1 cells treated with mASC total medium, mMVs depleted medium or mMVs rich medium from non-obese and non-diabetic patients. C) Healing rate line diagram of HMEC-1 cells treated with mASC total medium, mMVs depleted medium or mMVs rich medium from obese and non-diabetic patients. D) Healing rate line diagram of HMEC-1 cells treated with mASC total medium, mMVs depleted medium or mMVs rich medium from obese and diabetic patients (*P=0.05). Table S1. Adipogenic and osteogenic conditional medium composition. Table S2. Taqman gene expression assays. [file 13287_2019_1460_MOESM1_ESM.pptx]

## Slide 1
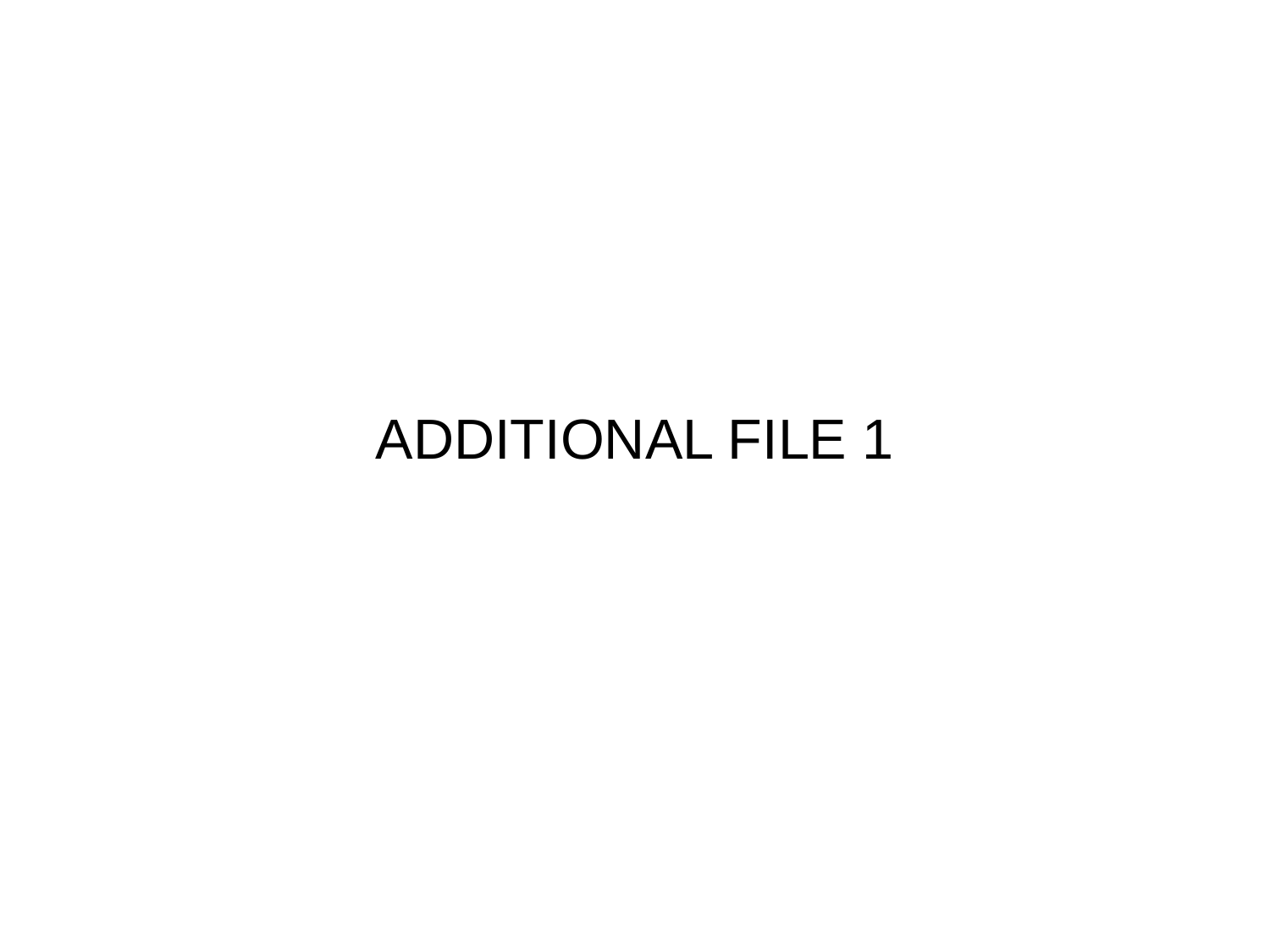

ADDITIONAL FILE 1

## Slide 2
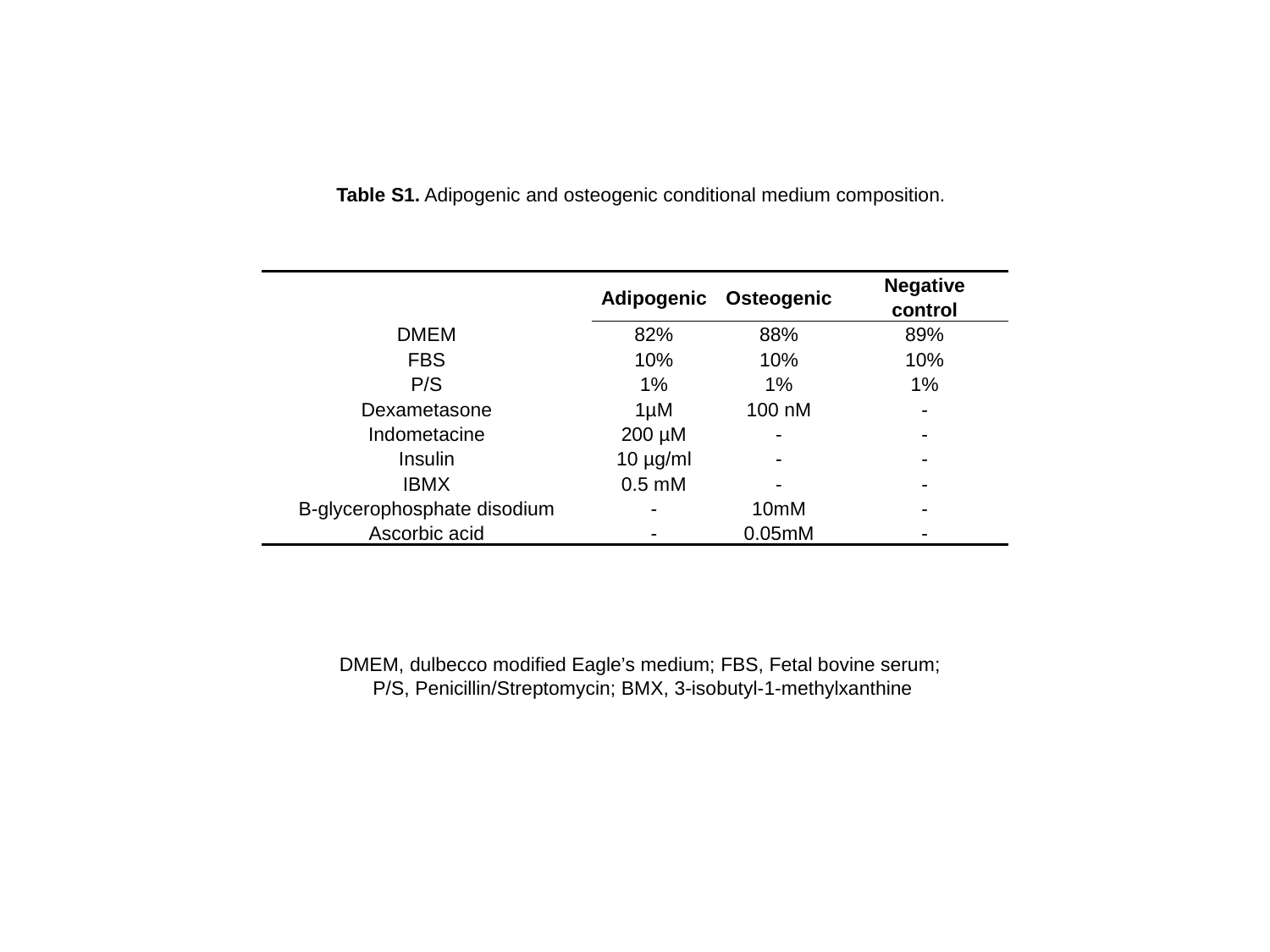

Table S1. Adipogenic and osteogenic conditional medium composition.
| | Adipogenic | Osteogenic | Negative control |
| --- | --- | --- | --- |
| DMEM | 82% | 88% | 89% |
| FBS | 10% | 10% | 10% |
| P/S | 1% | 1% | 1% |
| Dexametasone | 1µM | 100 nM | - |
| Indometacine | 200 µM | - | - |
| Insulin | 10 µg/ml | - | - |
| IBMX | 0.5 mM | - | - |
| Β-glycerophosphate disodium | - | 10mM | - |
| Ascorbic acid | - | 0.05mM | - |
DMEM, dulbecco modified Eagle’s medium; FBS, Fetal bovine serum;
 P/S, Penicillin/Streptomycin; BMX, 3-isobutyl-1-methylxanthine

## Slide 3
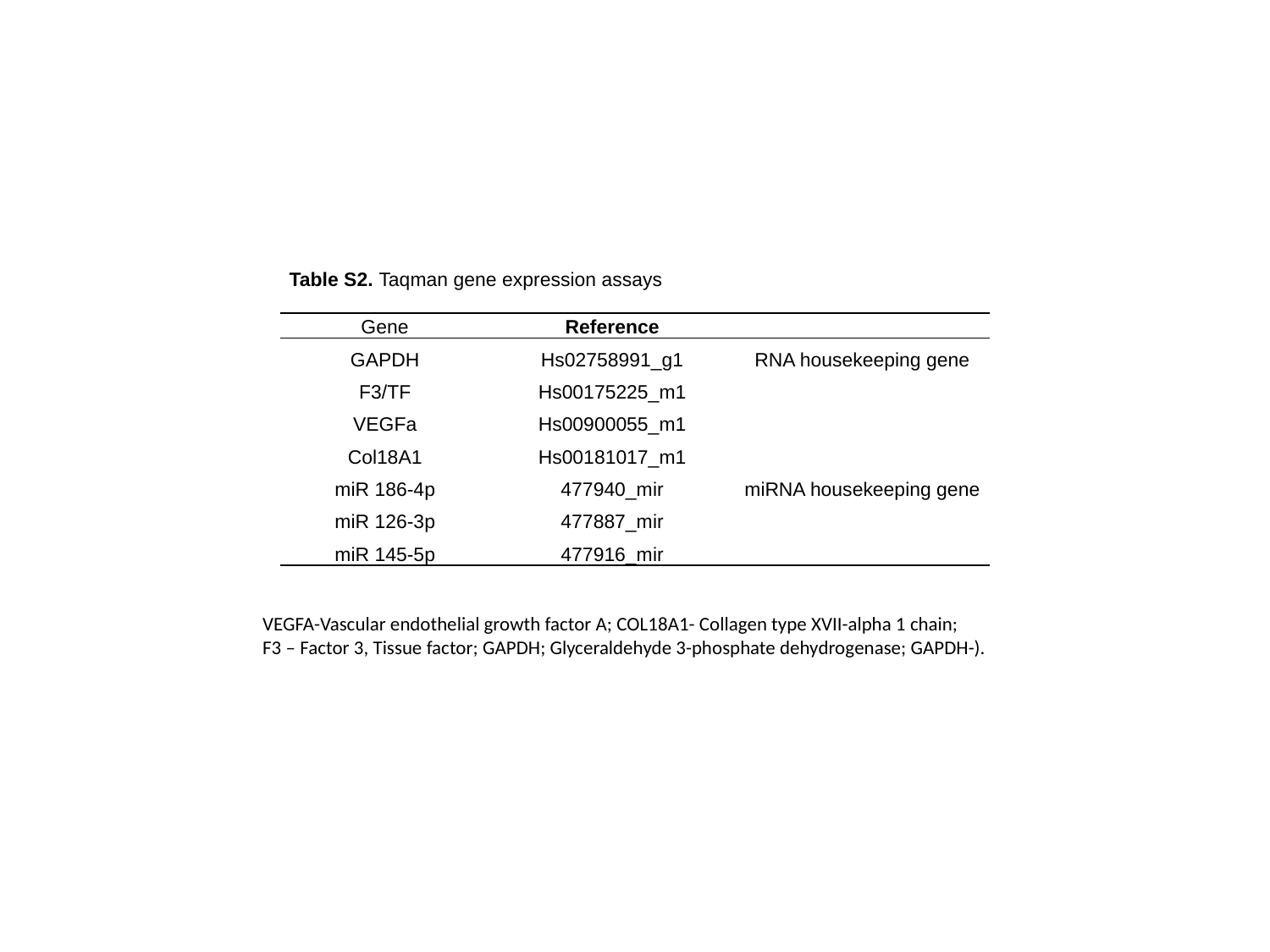

| Table S2. Taqman gene expression assays | | |
| --- | --- | --- |
| Gene | Reference | |
| GAPDH | Hs02758991\_g1 | RNA housekeeping gene |
| F3/TF | Hs00175225\_m1 | |
| VEGFa | Hs00900055\_m1 | |
| Col18A1 | Hs00181017\_m1 | |
| miR 186-4p | 477940\_mir | miRNA housekeeping gene |
| miR 126-3p | 477887\_mir | |
| miR 145-5p | 477916\_mir | |
VEGFA-Vascular endothelial growth factor A; COL18A1- Collagen type XVII-alpha 1 chain;
F3 – Factor 3, Tissue factor; GAPDH; Glyceraldehyde 3-phosphate dehydrogenase; GAPDH-).

## Slide 4
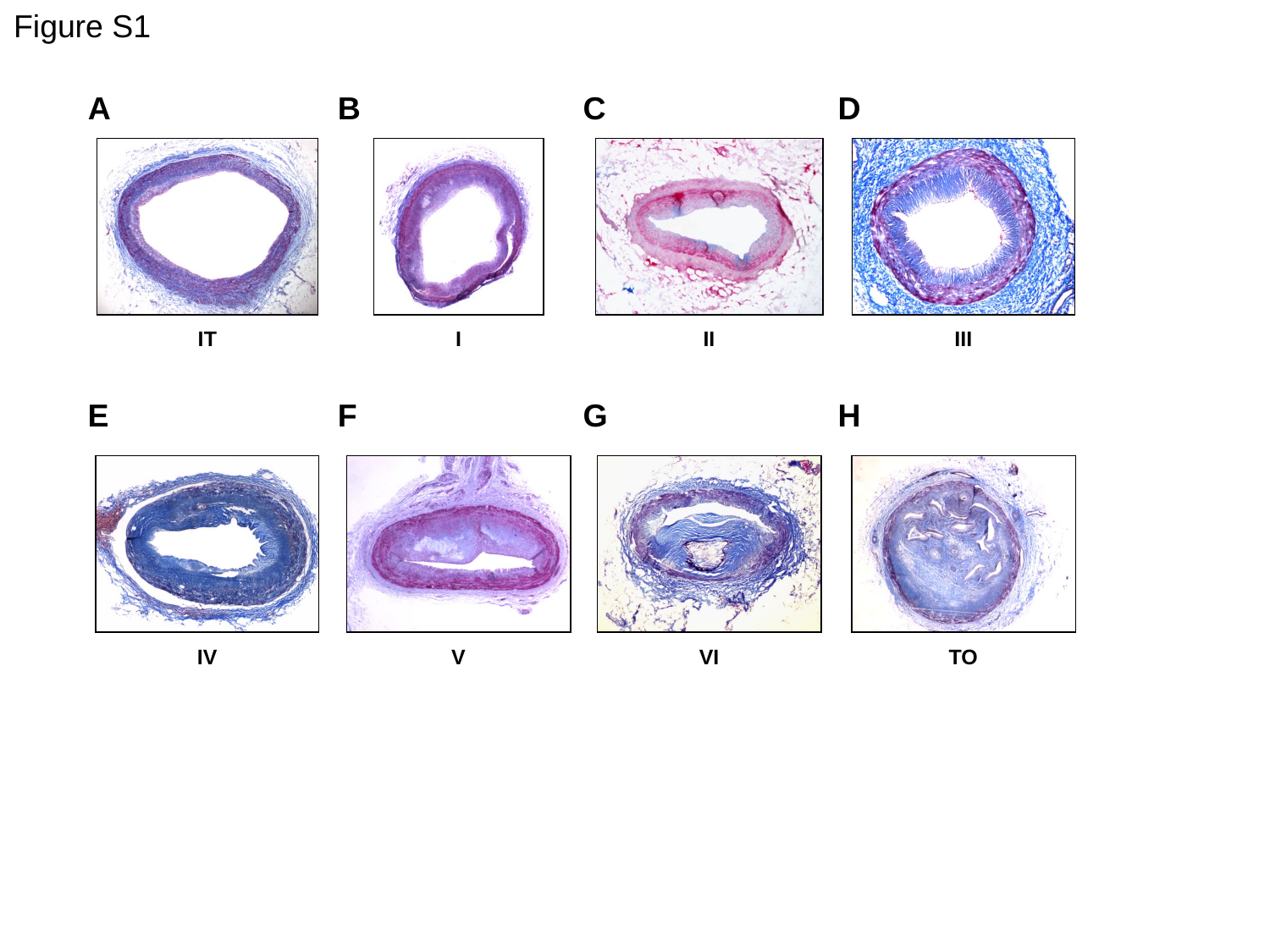

Figure S1
A
B
C
D
IT
I
II
III
E
F
G
H
IV
V
VI
TO

## Slide 5
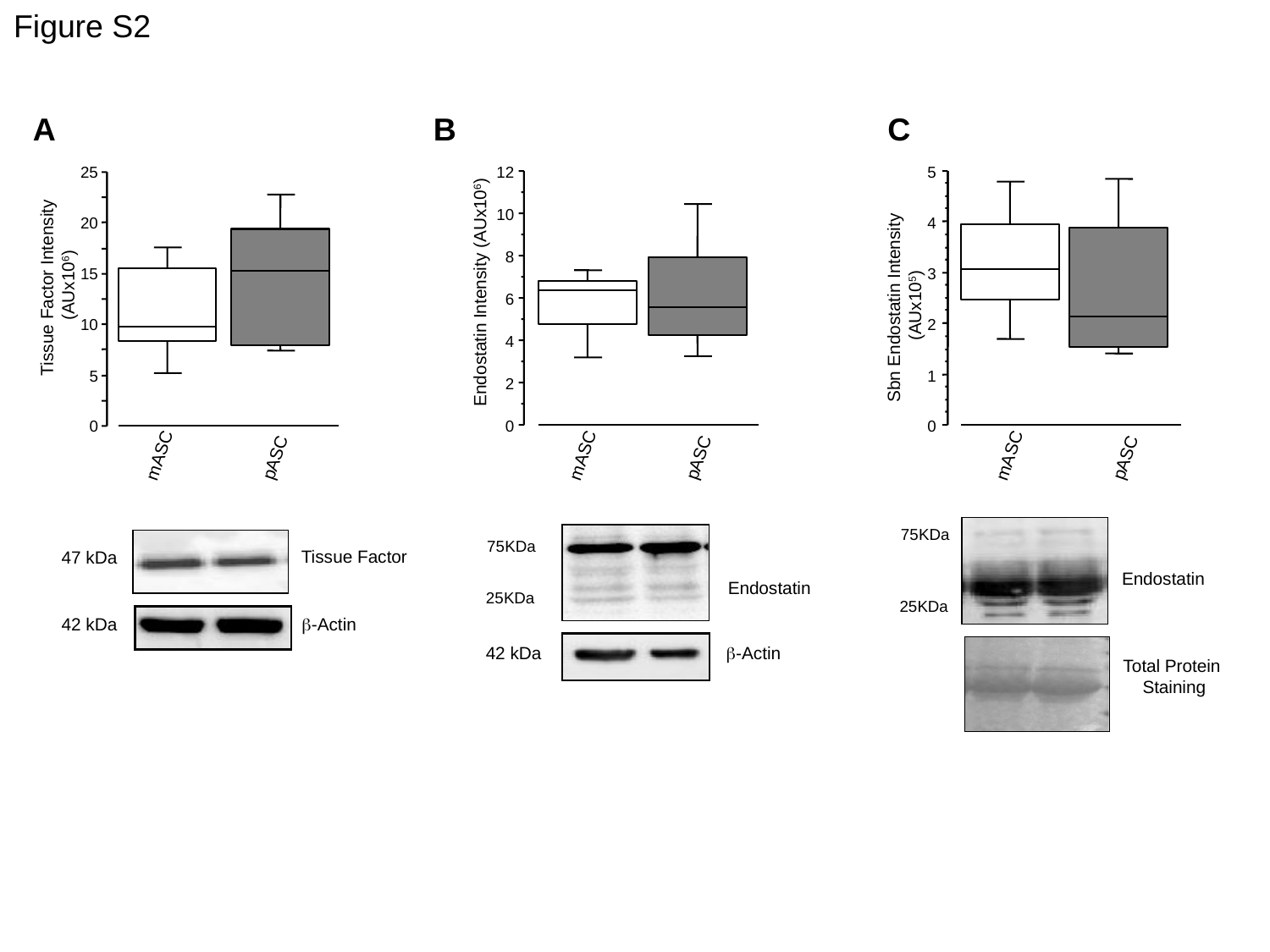

Figure S2
A
B
C
25
20
Tissue Factor Intensity
 (AUx106)
15
10
5
0
12
10
8
Endostatin Intensity (AUx106)
6
4
2
0
5
4
3
Sbn Endostatin Intensity
(AUx105)
2
1
0
mASC
pASC
mASC
pASC
mASC
pASC
75KDa
75KDa
Tissue Factor
47 kDa
Endostatin
Endostatin
25KDa
25KDa
42 kDa
b-Actin
42 kDa
b-Actin
Total Protein
Staining

## Slide 6
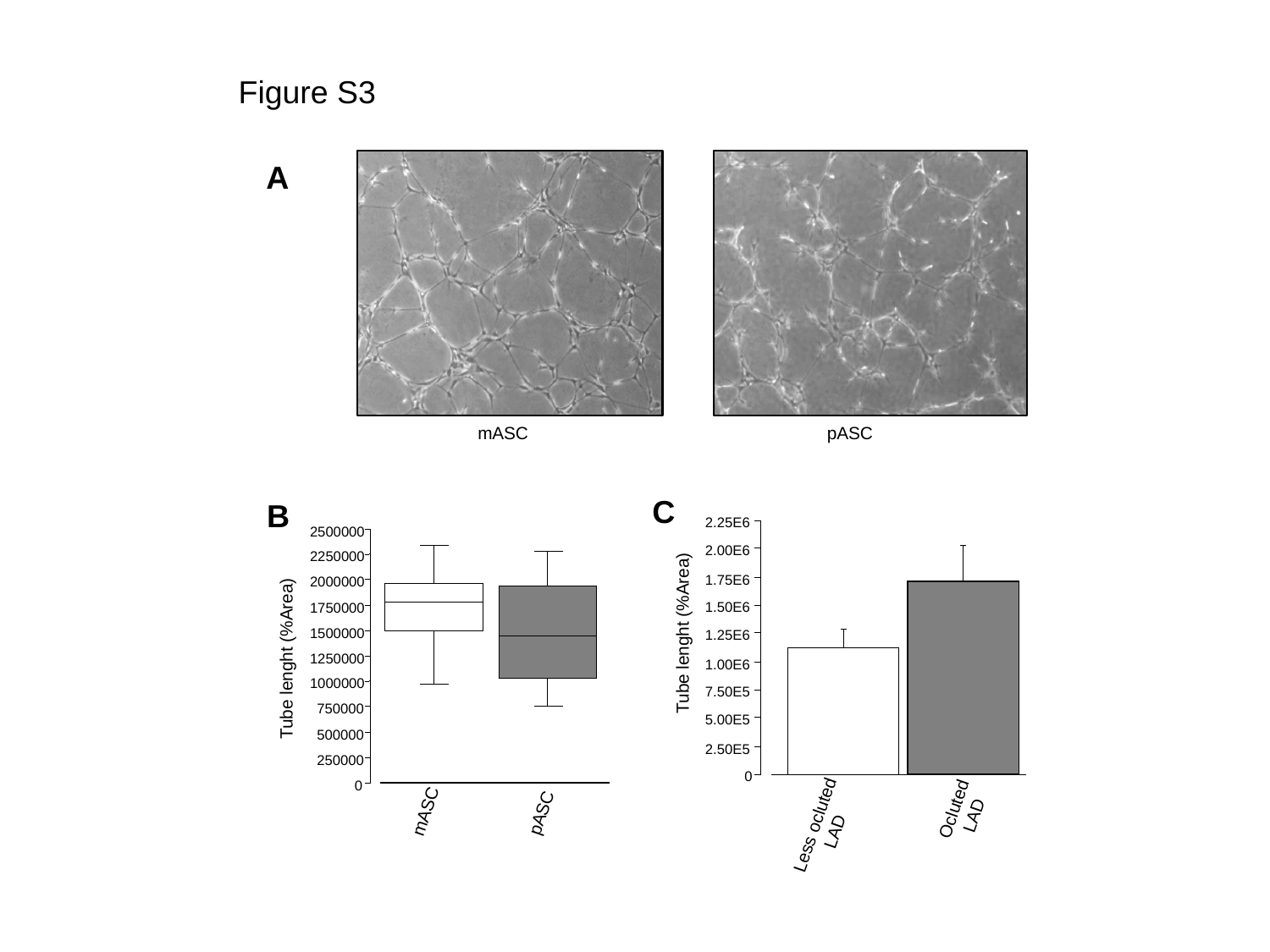

Figure S3
A
mASC
pASC
C
2.25E6
2.00E6
1.75E6
1.50E6
Tube lenght (%Area)
1.25E6
1.00E6
7.50E5
5.00E5
2.50E5
0
Ocluted
LAD
Less ocluted
LAD
B
2500000
2250000
2000000
1750000
1500000
Tube lenght (%Area)
1250000
1000000
750000
500000
250000
0
pASC
mASC

## Slide 7
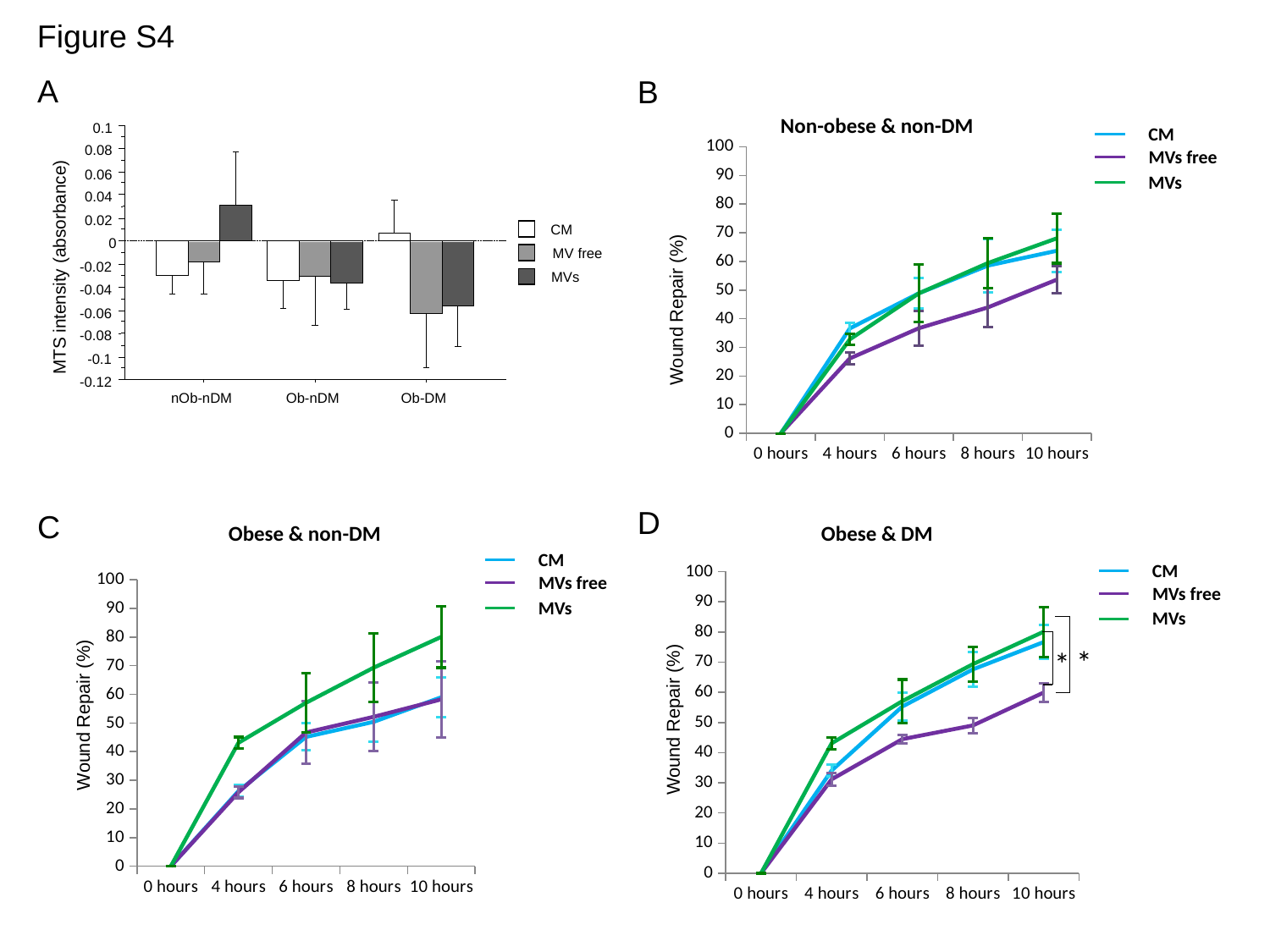

Figure S4
A
B
Non-obese & non-DM
CM
MVs free
MVs
0.1
0.08
0.06
0.04
0.02
CM
0
MV free
MTS intensity (absorbance)
-0.02
MVs
-0.04
-0.06
-0.08
-0.1
-0.12
nOb-nDM
Ob-nDM
Ob-DM
### Chart
| Category | 1nOb-nDM- CM | 1nOb-nDM - Mvfree | 1nOb-nDM - mvs |
|---|---|---|---|
| 0 hours | 0.0 | 0.0 | 0.0 |
| 4 hours | 36.5907115941336 | 26.129096025601893 | 32.78908283850985 |
| 6 hours | 48.8674922023802 | 36.6748243499466 | 48.84315630835574 |
| 8 hours | 58.53495164833412 | 43.94765940158779 | 59.401211086348745 |
| 10 hours | 63.70791366862568 | 53.632105332436275 | 68.06200811178562 |Wound Repair (%)
D
C
Obese & non-DM
Obese & DM
CM
MVs free
MVs
### Chart
| Category | 2Ob-nDM - CM | 2Ob-nDM - MVs free | 2Ob-nDM - MVs |
|---|---|---|---|
| 0 hours | 0.0 | 0.0 | 0.0 |
| 4 hours | 26.228535481275568 | 25.798103063340907 | 43.07215973023853 |
| 6 hours | 45.131849013931365 | 46.666086691695995 | 57.05532772101218 |
| 8 hours | 50.41423484931444 | 52.15769365242703 | 69.31674601181335 |
| 10 hours | 58.9897705004012 | 58.26556222742908 | 80.05161908478492 |CM
MVs free
MVs
### Chart
| Category | 3Ob-DM - CM | 3Ob-DM - MVs free | 3Ob-DM MVs |
|---|---|---|---|
| 0 hours | 0.0 | 0.0 | 0.0 |
| 4 hours | 34.07147571254511 | 31.1341585736846 | 43.07215973023853 |
| 6 hours | 55.28546814434925 | 44.45811782922447 | 57.05532772101218 |
| 8 hours | 67.59527142210648 | 49.01573095913683 | 69.31674601181335 |
| 10 hours | 76.62197881668861 | 59.903233826323756 | 80.05161908478492 |*
*
Wound Repair (%)
Wound Repair (%)
